# Bioinformation Informs the Allostasiome: Translational Environmental Restoration (TER) for the Climate Crisis Medical Emergency

**DOI:** 10.6026/97320630014446

**Published:** 2018-09-06

**Authors:** Francesco Chiappelli

**Affiliations:** 1 UCLA Center for the Health Sciences, Part-Time Faculty Biostatistics, CSUN, Department of the Health Sciences, Climate Reality Project Leadership Corps, International Research Consulting

**Keywords:** Medical emergency of the climate change crisis, Allostasis and the allostasiome, Micro-environment and macro-environment, Best evidence base (BEB), Translational Environmental Restoration (TER), bioinformation

## Abstract

Health care is optimized when the best evidence base (BEB) is translated into policies whose effectiveness can be verified. Bioinformation disseminates BEB and is critical to translational health care. The survival of all prokaryotes and eukaryotes, including mammals, and ultimately our species, depends upon their ability to adapt to changes in their micro-environmental milieu and to the challenges of their surrounding macro-environment. Disturbances in the organism's macro-environment, such as the stressful stimuli derived from environmental changes akin to the current climate crisis, alter its physiological, cytological, biological, epigenetic and molecular microenvironment, and trigger concerted allostatic responses to regain homeostasis. Individual patient data analysis advocates the allostasiome as the specific pattern of biological events and pathways each individual organism undergoes to regain a balanced state of homeostasis following macro-environmental insults. Translational Environmental Restoration (TER) is the translation of BEB in climate change research into effective and efficacious policies for restorative renewal of our macro-environment. Patient-centered translational health care in the current climate crisis depends upon defining and characterizing the allostasiome as a complex systemic process intertwined with TER. Bioinformation is timely and critical to climate crisis research in general and to TER specifically, because it informs and disseminates the best available evidence for each subject's allostasiome. Concerted research must define and characterize BEB of the multi-dimensional medical emergency produced by the current climate crisis. Novel lines of investigation, including allostasione research, increasingly depend on bioinformation for dissemination, and are foundational for TER, one plausible solution to this complex health care crisis.

## Bioinformation

Bioinformation (i.e., biological information) refers to the collection, analysis and interpretation of a range of physio-biological characteristics of an organism under study. Group and individual data from prokaryotes and sub-prokaryotic particles to eukaryotic organisms, from the lowest invertebrates to human subjects, are processed and disseminated as bioinformation. Bioinformation data may include genomic molecular fingerprints and physiological data (e.g., glycome), which together define, characterize, constitute and regulate the subject's microenvironment. Bioinformation findings also pertain to the situational environment (e.g., macroenvironment) in which the subject finds it, which has a determining impact upon the subject's physiological regulation. Bioinformation is the cornerstone of dissemination of new finding in the biomedical sciences. Therefore, Bioinformation is possibly the most timely and critical aspect of translational health care, which integrates the best evidence base (BEB) from translational research into treatment interventions that maximize effectiveness (i.e., translational effectiveness) [1].

Bioinformation is obtained by a variety of tools and instruments, from individual meetings to other traditional means of data sharing (e.g., scientific meetings, proceedings, press releases), and other contemporary modalities (i.e., internet, email, server farms, telehealth). The peer-reviewed written word remains the best approach the scientific community has to review and disseminate biomedical research findings, and to submit them to the rigorous evaluation designed to generate BEB. This journal, Bioinformation, has played a critical role in this process for over a decade, and continues to be as timely as it is critical to concerted progress and advances in biology in general and translational health care particularly.

## The Allostasiome

A common neologism in the scientific community, particularly in the biological and the medical sciences, is to add the suffix 'ome' to refer to the totality of a specific domain of study, exclusive of others. The origin of this terminology is traceable to German physician Dr. Hans Winkler, who defined, in 1920, the "das Genom" (i.e., the genome) as the haploid chromosome set "...which, together with the pertinent protoplasm, specifies the material foundation of the species..." [2]. Over the past decades, this terminology has expanded in translational research to the proteome, the interactome, the lipidome, the glycome, and many others. In the field of translational health care, we also speak of the bibliome as the specific set of reports considered for comparative effectiveness research, which have been rigorously screened to answer directly and specifically the PICOTS question [1].

We can expand this terminology even further in the context of study of the specific elements in the microenvironment that alter, for example the immune surveillance to certain tumors [3]. Similarly, those macro-environmental factors that powerfully disrupt the physiological balance (i.e., homeostasis) of the organism, such as immunotoxic factors, stress, and others, can be studied in this light. When severely disturbed, all organisms attempt to recover homeostatic balance. The concerted physiological, cellular and molecular events that are engaged in the process of regaining homeostasis are subsumed under the term of allostasis [4, 5]. The allostasiome is therefore defined as the set of conditions, events, processes and pathways that triggers the specific allostatic response of a given organism in response to micro- and macro-environmental changes. At the macro level, the gargantuan challenges are affected by climate crisi (e.g., heat stress, toxic gases, polluted waters) trigger significant allostasiomic adaptation.

## TER and allostasiome research of the next decade

Heat stress, breathable air and water - fresh and sea - acidity increase due to raised CO2 levels, clean water deprivation and drought, release of toxic gases are but a few of the consequences to the climate change crisis all living organisms are experiencing at every latitude. It matters not what the weather is in any given site on any given day: climate patterns have changed widely worldwide; and the situation is growing worse by the day. Experts do not speak of climate change any longer, but of climate crisis: a serious, aggressive, existential and extreme crisis that threatens all species - vegetal and animal, human beings included - and our planet as a whole. This is not an overstatement: climate data BEB convincingly show the precipitating downward pattern of life on earth as a direct consequence of climate change.

The macro-environmental stressors associated with climate change impact the disease-health trajectory of each individual patient. That is to say, the allostasiomic pattern of response to the variety of climate change-associated health threats is different and distinct in each individual patient. Consequently, to provide patient-centered health care, it is timely and critical to elucidate these allostasiomic patterns, and to disseminate this novel bioinformation. Concerted research on the systemic complexity of climate change informs policies directed at countering, salvaging and restoring our healthy survival on our planet. Translational Environmental Restoration (TER) refers to the translation of the best evidence base (BEB) in the climate change crisis, as one on the principal drivers of our macroenvironment, into effective and efficacious policies. The science of TER follows the same principles of finding BEB, translating BEB, testing the effectiveness of BEB and disseminating BEB as translational healthcare [1].

## Bioinformation for TER

The climate crisis causes serious threats to biological system, above and beyond the aforementioned macro-environmental stressors of heat challenge, de-oxygenation and carbon dioxide pollution of potable and sea water sources with consequential poisoning of planktons and fish, air particulates and their impact on depressing cellular immune surveillance to a variety of blood and solid tumors across vertebrate species, worsening lung disease, emphysema and asthmatic conditions, and psycho-cognitive and sleep disturbances. As serious is the outcome of the climate crisis on ocean and air current (e.g., jet stream, gulf stream), which pushes temperate climatic zone towards the pole. Consequently, the ice caps show dangerous melting patterns, and torrid humidity and heat spreading wider from the equatorial band. With that expansion of the tropical zones, mosquito-borne diseases spread wider and faster into heavily inhabited cities. Novel infectious diseases, including Ebola [6], Zika [7] and others [8] pose new challenges to public health and health care in Western societies. When making the case for patient-centered care [1] in this context, it behooves us to take into serious consideration the important role of the individual allostatic responses of each individual patient to the climatic macroenvironmental stressors discussed above. To be clear, patientcentered translational health care will only be achieved when the allostasiomic profile of each patient is systemically defined and characterized, and taken in account in the clinical treatment planning and delivery.

Bioinformation is timely and critical for translational health care. To be clear, bioinformation has a central role in TER as well because it informs the allostasiome, which is essential for patient-centered care and individual patient data research and analysis [9, 10]. Bioinformation plays an essential role in the concerted global action to address and control the complex and multi-dimensional medical emergency resulting from the climate change crisis [11, 12].

## References

[1] Chiappelli F. (2014). Springer-Verlag, Heidelberg.

[2] Winkler  H. (1920). Verlag Fischer, Jena..

[3] Oluwadara O (2011). Bioinformation..

[4] Chiappelli F, Cajulis OS. (2004). Quintessence Intern..

[5] Chiappelli F, Kutschman MM. (2018). NovaScience Publisher, Inc., Hauppauge, NY. Chapter 1,.

[6] Chiappelli F (2015). J Translational Med..

[7] Chiappelli F. (2014). Bioinformation..

[8] Chiappelli F. (2018). Bioinformation..

[9] Chiappelli F, Balenton N.  (2018). Translational Research Recent Progress and Future Directions, NovaScience Publisher, Inc., Hauppauge, NY.

[10] Khakshooy A, Chiappelli F. (2018). Springer-US, New York, NY.

[11] McMichael AJ (2003). World Health Organization, Geneva, ISBN 92 4 156248 X.

[12] Hathaway J, Maibach EW. (2018). Curr Environ Health Rep..

